# Design and construction of a servomechanism using a memory alloy linear actuator

**DOI:** 10.1016/j.ohx.2024.e00587

**Published:** 2024-09-27

**Authors:** Santiago Zuñiga, Diego Bravo, Carlos Rengifo

**Affiliations:** a Physics Department, Universidad del Cauca, Colombia; b Instrumentation and Control Department, Universidad del Cauca, Colombia

**Keywords:** Rotary joint, Servomechanism, Shape memory alloys, Prototype

## Abstract

This work shows the design and construction of a servomechanism of a rotator-type joint based on NiTi Shape Memory Alloys (SMA) with an angular position measurement based on a potentiometer sensor and digital electronic position control. The expected application of this prototype is for the use of small charges that emulate the movement of the human being, being bio-inspired and activated by artificial muscles, their potential applications they will be in medical and humanoid robotics. Computer Aided Design (CAD) allows evaluating and validating the most convenient parameters for construction of servomechanism, experimental results validate allowed us to obtain the values of the range of motion ±20° and a maximum torque of 1.01 kg-cm exerted on the axis of rotation for the prototype.

## Specifications table

**Table:** 

Hardware name	*Servomechanism using a Memory Alloy Linear Actuator.*
Subject area	•*Engineering and material science*
Hardware type	•*Servomechanism*
Closest commercial analog	*No commercial analog is available.*
Open source license	*CC BY 4.0*
Cost of hardware	*$ 124.108*
Source file repository	http://dx.doi.org/10.17632/jwzbvfj396.1

## 1 Hardware in context

The Shape Memory Alloy actuators (**SMA**), are commonly used in biomimetic robots due to their high power-to-mass ratio, inherent compliance, low noise levels, and light weight, [1]. SMA actuators allow the material to recover its initial state through internal or external heating stimuli. However, this type of actuators presents a high non-linearity and a parametric uncertainty and it is a challenge to develop a control strategy for this type of muscles based on SMA, [2].

The most widely used material for the manufacture of SMAs is the nickel–titanium alloy (NiTi) [3], [4], [5]. This type of actuators is widely used for robotics applications such as: medical robotics, self-configuring robots, biomimetic robots, robotic hands and manipulators and exoskeleton manufacturing, [6], [7].

The main objective of this document is to design and build a servomechanism using artificial muscles. To fulfill it, the mathematical model of the muscle–tendon must be determined in order to calculate the parameters of the CAD model necessary for the implementation of a prototype is shown in 1.

The energy recovery analysis is only possible in the case of actuators with elastic elements such as the one proposed in this work, unlike a conventional servomotor where the total energy is dissipated by the servomotor in the form of heat, [8]. The parameters obtained from the model allowed the design of a bioinspired actuator and the experimental results allow to validate its construction through rapid protyping using 3D printing.

The expected application of this prototype is for the use of small charges that emulate the movement of the human being, being bio-inspired and activated by artificial muscles, their potential applications they will be in medical and humanoid robotics, [7]. The main disadvantage compared to a commercial servomotor is the range of the angle of rotation, mainly due to the limited expansion of Nitinol (NiTi) (<8.5%) of the total length, [5]. The characteristics of the proposed prototype are summarized in Table 1.

**Fig. 1:**
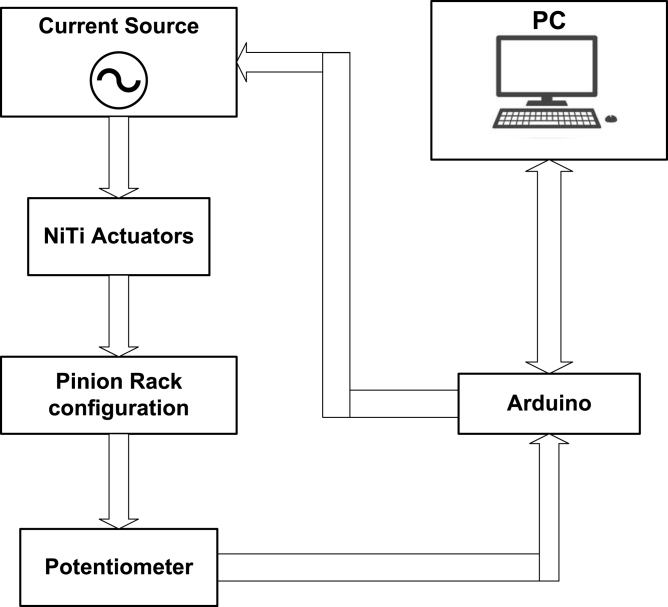
General scheme of a SMA servomechanism.

Mechanical systems actuated by Nitinol wires offer several advantages over those driven by electric motors. Nitinol, a shape memory alloy, possesses the remarkable ability to undergo significant deformation and recover its original shape upon heating. This unique property allows Nitinol wires to function as compact and efficient actuators in various mechanical applications. Unlike electric motors, Nitinol-based systems have no wearing parts such as bearings or gears, allowing for long life. Additionally, the inherent flexibility and lightweight nature of Nitinol wires enable their integration into intricate designs with fewer space constraints compared to bulkier electric motors. This advantage is especially beneficial in applications requiring precise and delicate movements, such as medical devices or micro-robotics. Another advantage is that Nitinol actuators operate silently, devoid of the noise and vibrations generated by electric motors, making them well-suited for noise-sensitive environments.

**Table 1:** Servo characteristics.

Weight	120 g
Dimensions	85×65×40 mm
Torque	1.01 kg-cm
Rotation	±20°
Control signal	Digital

The article is structured as follows: Section 2 describes the hardware implemented, Section 3 is dedicated to the design files and Section 5 presents the build instructions necessary for the construction and right operation of the device, followed by the validation and characterization presented in Section 7 and ending with the conclusion of the article in Section 8.

## 2 Hardware description

**Fig. 2:**
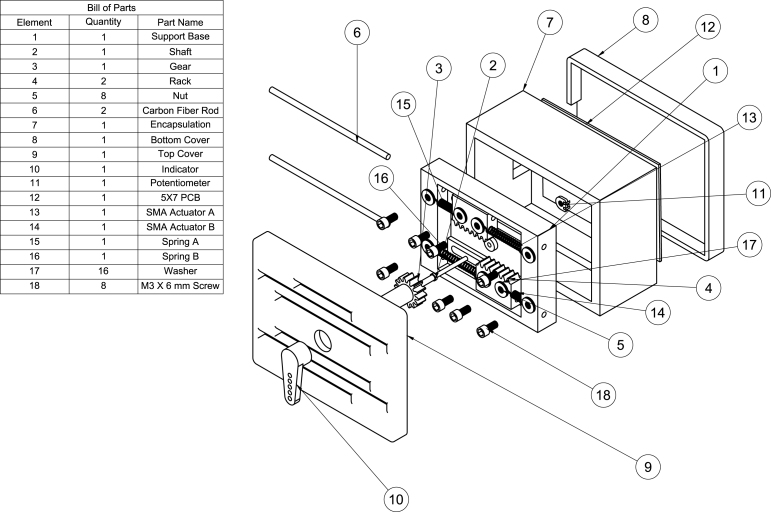
Scheme of the proposed mechanic system.

**Fig. 3:**
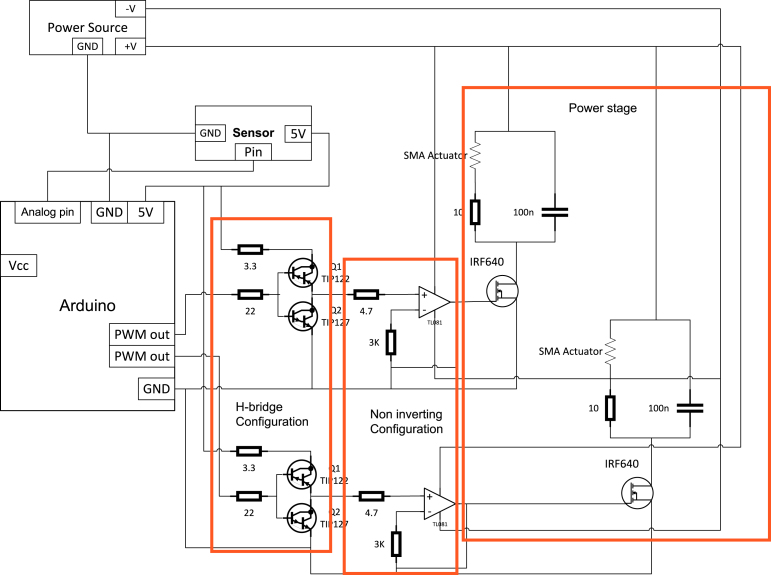
Scheme of the proposed electronic system.

The proposed servomechanism system consists of the following elements: (i) a rack and pinion configuration composed of two SMA actuators responsible for generating the necessary pulling force for the rotation of the main shaft. Additionally, it is composed of two springs with dimensions close to the actuators, whose spring constant does not exceed 150 N/m. These are responsible for bringing them to their initial stage (Fig. 2); (ii) an electronic switching plant that regulates the induced current in the actuators by varying the input PWM signal; (iii) a PT-15 carbon potentiometer from the PIHER brand, which will function as a transducer and will convert the angular signal received by the pinion into an electrical signal, which will be used for calculating the control signal of the system; (iv) an Arduino Mega 2560 board that will receive the signal from the potentiometer through the analog input and will perform the control signal calculations in order to send a PWM signal at different frequencies to obtain the desired position signal. Fig. 3 shows a more detailed scheme of the connections for the proper functioning of the servomechanism.

### 2.1 Power stage

The power stage connected to the output of the operational amplifier is used to drive a load, such as a motor, actuator, or speaker. In this particular configuration, the MOSFET’s gate is connected to the amplifier’s output, the source is grounded, and the drain is connected in parallel with a capacitor and an SMA actuator in series with a 10 Ω, 5-W resistor. This resistor serves the purpose of preventing overheating in the SMA actuator, and its value was experimentally selected to ensure the proper functioning of the system. This circuit also requires a power supply to provide the necessary voltage for driving the load. The role of the power stage is to amplify and deliver the amplifier’s output voltage to the load while also providing protection against overloads or short circuits. The combination of the capacitor and resistor in series with the load is used to filter any high-frequency noise or ripple that may be present in the output signal, while the SMA actuator converts the electrical signal into mechanical motion.

The voltage gain of the non-inverting amplifier configuration, which is used as the input for the power stage, is given by Eq. (1), where $V_{out}$ is the output voltage, $V_{in}$ is the input voltage from the operational amplifier, $R_{f}$ is the feedback resistor, and $R_{1}$ is the input resistor [9].

(1) $$V_{out}=V_{in}\left(1+\frac{R_{f}}{R_{g}}\right)\;\left(\text{V}\right)$$

The drain current ($I_{D}$) of the MOSFET can be calculated using the equation:

(2) $$I_{D}=\frac{k{\left(V_{GS}-V_{th}\right)}^{2}}{2}\;\left(\text{A}\right)$$

where k is the MOSFET transconductance parameter, $V_{GS}$ is the gate–source voltage, and $V_{th}$ is the threshold voltage.

The voltage across the load can be calculated using the voltage divider equation:

(3) $$V_{load}=V_{out}\left(\frac{R_{L}}{R_{L}+R_{D}}\right)\;\left(\text{V}\right)$$

where $V_{load}$ is the voltage across the load, $R_{L}$ is the resistance of the load, and $R_{D}$ is the drain resistor.

The time constant of the RC filter can be calculated using the equation:

(4) $$\tau =R_{L}\cdot C\;\left(\text{s}\right)$$

where τ is the time constant, $R_{L}$ is the resistance of the load, and C is the capacitance of the capacitor. The capacitor and resistor combination in series with the load are used to filter any high-frequency noise or ripple that may be present in the output signal.

The displacement of the SMA actuator can be calculated using the equation:

(5) $$x=\frac{F}{k_{s}}\;\left(\text{m}\right)$$

where x is the displacement of the SMA actuator, F is the force applied to the actuator, and $k_{s}$ is the spring constant of the actuator. The SMA actuator converts the electrical signal into mechanical motion to drive the load.

Overall, the power stage amplifies and delivers the output voltage of the operational amplifier to the load while providing protection against overloading or short circuits. The equations provided above are critical for understanding the behavior and performance of the power stage [10].

#### 2.1.1 Transistor heating solution

The solution to address transistor overheating in our setup involved the use of heat sinks and thermal paste. These components played a pivotal role in effectively managing the transistor temperatures. Heat sinks provided a larger surface area for heat dissipation, while thermal paste improved the heat transfer between the transistors and the heat sinks. This combination of measures resulted in a significant reduction in transistor temperatures, ensuring the safe and stable operation of our configuration.

### 2.2 Manufacturing and characterization of the actuator

To construct the actuator, the process was divided into two stages. The first stage involved an initial heating to achieve the adaptation of the Nitinol SMA wire. The second stage encompassed the training phase to ensure that the actuator retains the desired shape.

Table 2 presents the temperature values and duration for each stage. In the first stage, we shape the actuator using a screw to create a Nitinol spring, followed by heating it to 350 °C for 120 min. This temperature was selected because it allows the material to adapt without inducing restructuring. Choosing a lower temperature would not have allowed for the proper formation of the desired structure, while a higher temperature could have caused premature material restructuring, which would not be desirable for the process.

**Table 2:** Temperature and duration data.

Stage	Heating (℃)	Cooling (℃)	Duration (min)
First stage	350	24	120
Second stage	600	24	720

In the second stage, we heat the Nitinol SMA wire to 600 °C for 720 min (12 h) to achieve a more profound and lasting material adaptation. This specific temperature is based on the thermomechanical properties of the material. Heating to this temperature for this extended period allows for a more complete material adaptation, which is essential to achieve the desired final shape of the actuator. After completing this stage, the actuator is cleaned using isopropyl alcohol to remove residues from the heating process. This cleaning step is necessary to ensure a clean final product free from any residues that could affect its performance.

In summary, the choice of these specific temperature degrees is based on the need to balance the formation of the desired structure with the prevention of premature material restructuring. These temperatures were determined based on previous research and technical considerations and represent an optimal compromise for the process, while the alcohol cleaning step ensures the integrity of the final product.

In Table 3, the physical characteristics of the actuator will be presented.

The alloy’s density is elevated due to its composition, consisting of approximately 50% nickel. The addition of titanium decreases the density to 6450 kg/$m^{3}$, which is close to that of steel (7850 kg/$m^{3}$). An alloy SMA wire with a maximum diameter of 0.375 mm and lengths not exceeding 45 cm was employed for the initial prototype tests. By utilizing the manufacturer-provided data, the actuator’s weight can be calculated.

(6) $$A=\pi \frac{d^{2}}{4}=\pi {\left(\frac{0.375^{-3}}{2}\right)}^{2}\;{\text{m}}^{2}=1.1045\;{\text{m}}^{2}$$

$$V=L\cdot A=0.45\;\text{m}\times 1.1045\;{\text{m}}^{2}=4.97025\times 10^{-8}\;{\text{m}}^{3}$$

$$\text{mass}=\rho \cdot V=4.97025\times 10^{-8}\;{\text{m}}^{3}\times 6450\;{\text{kg/m}}^{3}=3.2\times 10^{-4}\;\text{kg}=0.32\;\text{g}$$

where: L is the SMA wire length, A is the SMA wire cross-sectional area, d is the SMA wire diameter and ρ is the alloy density. The weight can be considered negligible for the system as the goal is to have a prototype with reduced weight and dimensions. The thermal conductivity of the alloy is 18 W/m°C, similar to that of stainless steel (14–15 W/m°C). Given the material’s capacity to conduct heat, it can be considered quite good. During the heating stage, a good actuation speed can be achieved, allowing temperatures of 100 °C to be reached in a few seconds by applying sufficiently large current spikes.

**Table 3:** Physical properties of the actuator.

Properties	Value
SMA wire length (mm)	450
Compressed spring (mm)	19
Spring diameter (mm)	4

Another crucial aspect to consider is the cooling time of the Nitinol SMA wire. This time is directly related to the force it produces and its diameter. In this case, a larger diameter SMA wire was used to achieve greater force during the austenitic transformation, resulting in a cooling time exceeding 10 s, considered slow cooling that leads to a slower dynamic response. To address this issue, the manufacturer suggests using a junction with a material of high thermal conductivity to achieve better cooling. The electrical resistance of the alloy is very low, on the order of micro ohms. The 0.375 mm Nitinol SMA wire can handle a nominal value of 2750 A of direct current, but once this current limit is exceeded, the material overheats and breaks within a few seconds. A low electrical resistance ensures reduced power consumption, less than 30 W, as demonstrated below:

(7) $$P=I^{2}\cdot R^{\prime }\cdot L$$

$$P={\left(2.750\;\text{A}\right)}^{2}\cdot \left(8\;\Omega /\text{m}\right)\cdot \left(0.45\;\text{m}\right)=27.225\;\text{W}$$

Being: I is the rated current provided by the manufacturer, with a maximum of 2.75 A, $R^{\prime }$ the SMA wire resistivity, 0.375 mm diameter (8 Ω/m) and L the SMA wire length (max 45 cm).

**Fig. 4:**
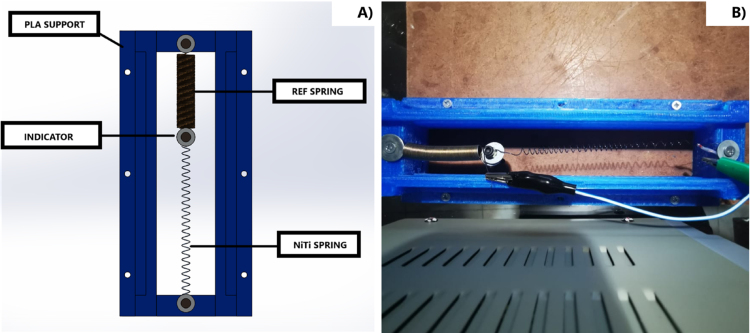
Experimental setup for SMA characterization. (a) Side view (b) Top view.

The material was characterized using an empirical yet robust method, involving maintaining a constant voltage while varying current levels to observe the deformation exhibited by the material under different temperature conditions. A well-designed base was employed for this purpose, securing the actuator along with a reference spring and an indicator to facilitate the analysis of the system’s trajectory. This setup can be observed in Fig. 4. The characterization process was divided into four essential stages.

**Fig. 5:**
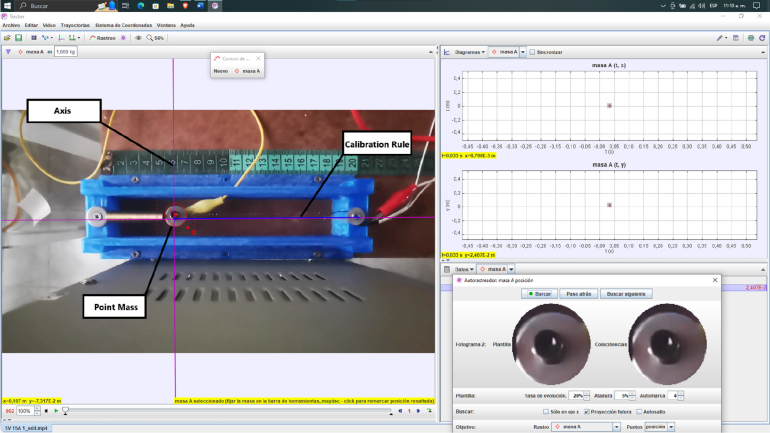
Tracker software.

• Step 1: The actuator was subjected to various current values (0.7, 1, 1.3, 1.5, 1.7, 1.9 A) at a fixed voltage of 5 V. The selection of these current values was based on specific considerations: the current of 0.7 A was chosen as a starting point since it is the minimum current required for the actuator to operate effectively, while the current of 1.9 A represents the maximum current achievable due to the limitations of the voltage source used. This range of current values allowed us to assess the actuator’s performance across a spectrum ranging from its operational threshold to its maximum capacity, providing significant insights into its behavior under varying loads. Each current value was repeated three times for each temperature value, with a duration of 60 s per sample, to ensure the collection of reliable and representative data. During this process, temperature data was obtained using a 100k NTC thermistor, while deformation data was recorded through data capture. The choice of a nominal current of 1.9 A instead of 2.75 A is based on the available voltage source’s capacity, which was limited to 2 A. This was essential to maintain the integrity of the components and ensure the safety and stability of the process. Exceeding the nominal capacity of the voltage source could have resulted in unstable operation, excessive heating, or even damage to the voltage source. Additionally, this choice allowed for the replication of realistic conditions and ensured the repeatability and consistency of results in future experiments.
• Step 2: The software “Tracker Video Analysis and Modeling Tool” was utilized for video processing, involving the calibration of the video’s first frame. In this process, three key elements were established: the axis of system motion, the configuration of a calibration rule necessary for the program to generate motion graphics, and, finally, the creation of a point mass that will create a precise mask delimiting the object of analysis. These procedures are illustrated in Fig. 5.
• Step 3: After setting up the marker, reference frames were fixed to analyze each frame and extract the indicator’s position in each of them.
• Step 4: Lastly, displacement data is graphed, and parameters such as velocity, acceleration, momentum, and kinetic energy of the system are abstracted from the displacement data.

The process yielded the data presented in Table 4, these parameters being of significant importance for the control system’s development.

This characterization method is valuable for open-loop control system development. As the force generated by these types of actuators is comparable to that of micromotors, it proves advantageous for lighter system development.

**Table 4:** Characterization data.

Current (A) - Voltage (V)	Deformation (cm)	Temperature (℃)	Force (N)
0.7–5	1.71	35.09	0.87
1.0–5	2.02	50.95	1.03
1.3–5	2.20	59.42	1.13
1.5–5	2.33	68.72	1.19
1.7–5	2.56	74.93	1.31
1.9–5	2.54	83.91	1.30

**Fig. 6:**
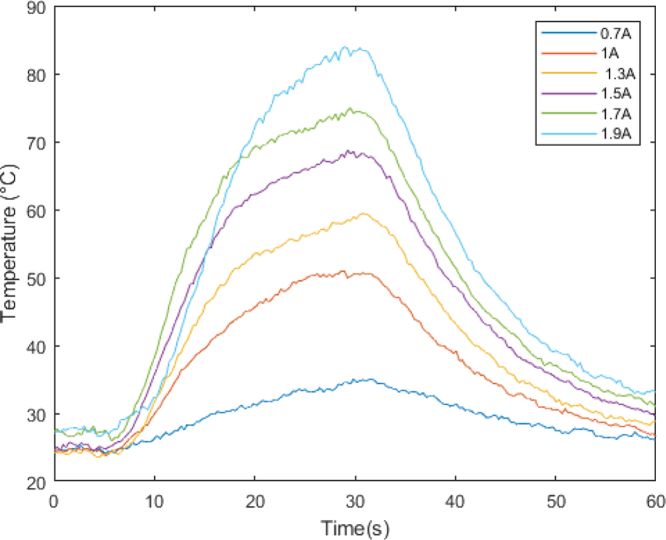
Temperature variation over time.

As previously discussed, one of the most significant challenges for these types of actuators revolves around cooling time. These actuators exhibit sluggish operation at room temperature, as illustrated in Fig. 6. Within the context of this research, a proposed solution involves employing an agonist–antagonist configuration to enhance the actuator’s cooling process. This configuration enables the actuator to transition effectively from the Austenite phase to an organized Martensite phase, all facilitated by the intervention of an external force.

By observing Fig. 6, we can clearly see how the material experiences a much more pronounced temperature increase as the current is raised, reaching its highest point at a current of 1.9 A. This behavior suggests that the actuator operates effectively by raising the temperature in tandem with the increasing current. This phenomenon, in turn, directly influences key parameters such as the force and deformation of the Nitinol material.

**Fig. 7:**
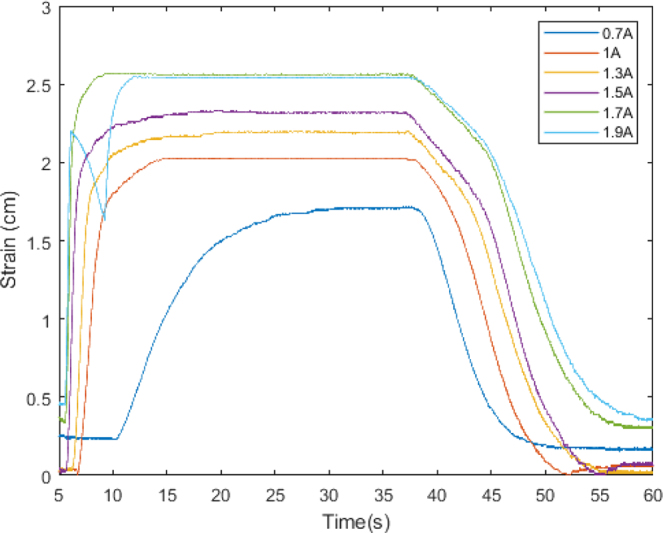
Strain variation over time.

**Fig. 8:**
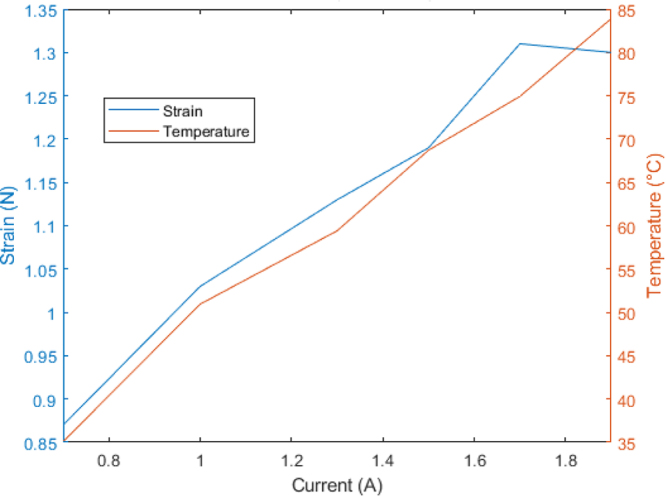
Correlation of strain, temperature, and current.

As evident in Fig. 7, the actuator’s behavior aligns with descriptions found in the literature. Examining the curve corresponding to 0.7 A, it becomes apparent that deformation will be comparatively lower than at higher current levels. This phenomenon arises from the fact that the material’s exerted force maintains a direct proportionality to the temperature value, a correlation depicted more comprehensively in Fig. 8.

### 2.3 Rack and pinion configuration

To achieve the agonist–antagonist muscle configuration in a robotic joint, a rack and pinion configuration is established in parallel. The activation method for each of the racks is an SMA actuator, and the restoration system is a spring.

The SMA actuators are connected in parallel, which generates a positive movement when one actuator is activated and a negative movement when the other is activated. The linear movement of the racks is translated into angular movement of the pinion, thereby generating the characteristic movement of a joint.

This configuration allows for better control and coordination of the robotic joint movement, mimicking the natural motion of a human joint. Additionally, the use of SMA actuators provides an efficient and lightweight solution for actuation, as they require low power and can easily be integrated into the robotic joint design.

Overall, the rack and pinion configuration with SMA actuators provides a promising solution for achieving agonist-antagonist muscle configuration in robotic joints, contributing to the development of more advanced and human-like robotic systems, [8].

**Fig. 9:**
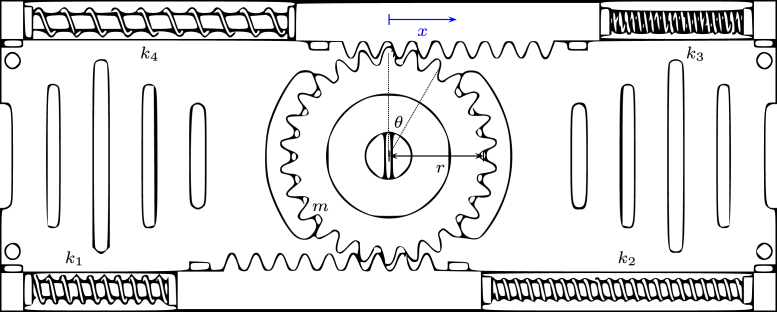
Diagram of the servo mechanism.

The Fig. 9 shows a servo mechanism actuated by artificial muscles. The physical parameters are the spring constants $k_{1}$, $k_{2}$, $k_{3}$ and $k_{4}$, r is the radius of the pinion, m is the mass, J is the moment of inertia about the axis, and θ is the rotation angle. The translational motion is denoted by the displacement x=r⋅θ.

To obtain the mathematical model, the friction between the rack–pinion drive is not considered so that the total mechanical energy is conserved [8]. The kinetic energy T and potential energy U of the dynamic system of Fig. 9 are:

(8) $$T=\frac{1}{2}m{\overset{˙}{x}}^{2}+\frac{1}{2}J{\overset{˙}{\theta }}^{2}=\frac{1}{2}mr^{2}{\overset{˙}{\theta }}^{2}+\frac{1}{2}J{\overset{˙}{\theta }}^{2}=\frac{1}{2}\left(J+mr^{2}\right)\;{\overset{˙}{\theta }}^{2}\;\left(\text{J}\right)$$

$$U=\frac{1}{2}k_{eq}x^{2}=\frac{1}{2}k_{eq}r^{2}\theta^{2}\;\left(\text{J}\right)$$

where $k_{eq}=k_{1}+k_{2}+k_{3}+k_{4}$ since the springs are in parallel. In a conservative system.

(9) $$\frac{d}{d\;t}\left(T+U\right)=0\left(J+mr^{2}\right)\;\overset{˙}{\theta }\;\overset{¨}{\theta }+k_{eq}r^{2}\;\theta \;\overset{˙}{\theta }=0\left(J+mr^{2}\right)\;\overset{¨}{\theta }+k_{eq}r^{2}\;\theta =0$$

The second-order differential equation that represents the servo mechanism of Fig. 9 is:

(10) $$\overset{¨}{\theta }\left(t\right)+\frac{k_{eq}r^{2}}{J+mr^{2}},\theta \left(t\right)=0$$

The natural frequency $\omega_{n}$ of the system is obtained from Eq. (10):

$$\omega_{n}=\sqrt{\frac{k_{eq}r^{2}}{J+mr^{2}}}\;\text{[rad/s]}$$

The combination of mechanical and electronic systems is crucial to the construction of a servomechanism based on SMA actuators. The precision and responsiveness of the mechanical system, together with the monitoring and control capabilities of the electronic system, enable the servomechanism to precisely control the position and force of the SMA actuator. This allows the system to perform complex tasks that would require extreme precision and skill if done manually. Integrating these systems also enables the servomechanism to be used in applications where precision and speed are critical, such as in robotics, manufacturing, and medicine. In summary, the combination of mechanical and electronic systems is essential for the construction and operation of a servomechanism based on SMA actuators.

**Fig. 10:**
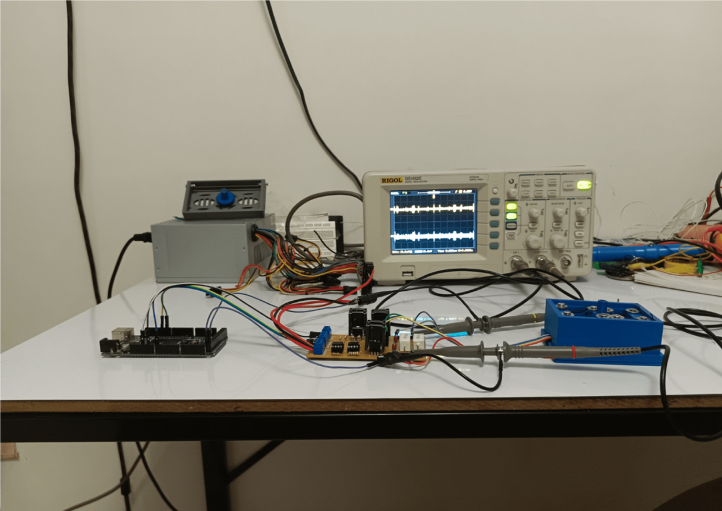
Servomechanism based on SMA actuator.

### 2.4 Control system

The servomechanism represented by the Eq. (10) can be stabilized by the Proportional–Integral–Derivative (PID) feedback control law.

(11) $$I\left(s\right)=-k_{p}\;\Theta \left(s\right)+\frac{k_{p}}{T_{i}\;s}\left(\Theta_{d}\left(s\right)-\Theta \left(s\right)\right)-\frac{k_{p}\;T_{d}\;s}{\frac{Td}{N}s+1}\Theta \left(s\right)$$

**Fig. 11:**
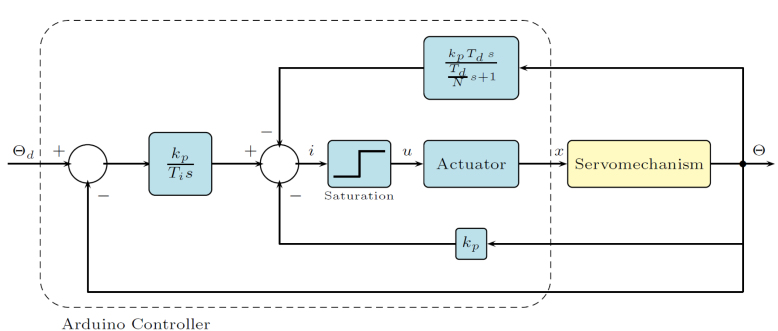
Block diagram of PID control.

$k_{p}$ is the proportional gain, $T_{i}$ is the integral time, $T_{d}$ is the derivative time and $\frac{1}{\left(T_{d}/N\right)\;s+1}$ is a low-pass filter to avoid high-frequency measurement noise. Eq. (11) is called *velocity PID algorithm*, [11]. It has the advantages of bumpless transfer and less reset windup 0 or 100% and are better suited for controlling servo motor-driven devices. The control law Eq. (11) specifies the current i over the actuator (Nitinol) that moves a distance x. Fig. 11 shows the control system implemented.

**Fig. 12:**
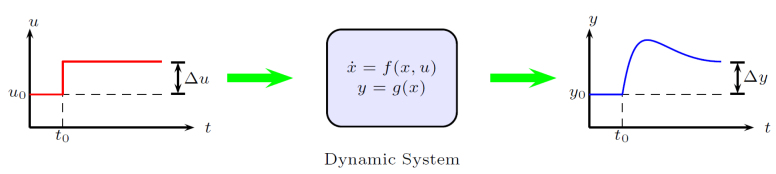
Tuning method for the PID.

Controller was sintonized using trial and error from simulation model until to obtain satisfied results. Fig. 12 illustrates the method of tuning the controller around the operating point $\left(u_{0},\;y_{0}\right)$ of the servomechanism [12], a small voltage amplitude step signal ΔU is injected into the servomechanism and the output response y, in this case, a small rotation angle, allows us to find an empirical model of the system $G\left(s\right)=\frac{\Delta y\left(s\right)}{\Delta u\left(s\right)}$ that allows adjusting the controller parameters.

The controller parameters that allowed to obtain the best result were $k_{p}=150$, $T_{i}=30$ and $T_{d}=50$. The sample time is h=0.01 s. Test and validation graphs of the implemented controller are shown in Section 7. The following section presents the electronic and mechanical design files of the servomechanism.

## 3 Design files

The following table shows the figures that correspond to design of the proposed Servomechanism based on SMA Actuator.

**Table:** 

Design file name	File type	Open source license	Location of the file
General scheme of a Servomechanism	Figure (PNG)	CC BY 4.0	Included in the article (Fig. 1)
Scheme of the proposed mechanic system	Figure (PNG)	CC BY 4.0	Included in the article (Fig. 2)
Scheme of the proposed electronic system	Figure (PNG)	CC BY 4.0	Included in the article (Fig. 3)
H-bridge configuration	Figure (PNG)	CC BY 4.0	Included in the article (Fig. 3)
Non-inverting configuration	Figure (PNG)	CC BY 4.0	Included in the article (Fig. 3)
Diagram of the servomechanism	Figure (PNG)	CC BY 4.0	Included in the article (Fig. 9)
Servomechanism based on SMA actuator	Figure (PNG)	CC BY 4.0	Included in the article (Fig. 10)
Manufactured SMA actuator	Figure (PNG)	CC BY 4.0	Included in the article (Fig. 13)
Construction plans	Figure (PNG)	CC BY 4.0	Included in the article (Fig. 14)
Control plant schematic	Figure (PNG)	CC BY 4.0	Included in the article (Fig. 15a)
Control plant PCB file	Figure (PNG)	CC BY 4.0	Included in the article (Fig. 15b)
Connection SMA schematic	Figure (PNG)	CC BY 4.0	Included in the article (Fig. 15c)
Connection SMA PCB file	Figure (PNG)	CC BY 4.0	Included in the article (Fig. 15d)
Manufactured PCB	Figure (PNG)	CC BY 4.0	Included in the article (Fig. 15e)

Files corresponding to the design and programming of the Servomechanism are in the Servomechanism System folder of the repository: http://dx.doi.org/10.17632/jwzbvfj396.1

• **Code:** This folder contains files corresponding to Arduino Scripts used for PID Control(file FINAL.ino).
• **Design Files:** This folder contains files corresponding to the design of the servomechanism.
• **Gerber Files:** This folder contains files corresponding to Gerber Files to manufactured the PCBs.
• **Schematics:** This folder contains files corresponding to the electronic design of the control plant.
• **STL Files:** This folder contains files corresponding to the STL files for manufacturing the 3D pieces of the servomechanism.

## 4 Bill of materials summary

The list of materials used in the design of the Servomechanism are presented in the following table.

**Table:** 

Designator	Component	Number	Cost per unit-currency	Total cost-currency	Source of materials	Material type
SMA Actuator	Flexinol Wire 0.015” LT	1	$ 42.25	$ 42.25	Robotshop	Metal
Resistor	$3.3\;\Omega ,\frac{1}{4},W$	2	$ 0.022	$ 0.044	Mercadolibre	Other
Resistor	$4.7\;\Omega ,\frac{1}{4},W$	2	$ 0.022	$ 0.044	Mercadolibre	Other
Resistor	$22\;\Omega ,\frac{1}{4},W$	2	$ 0.022	$ 0.044	Mercadolibre	Other
Resistor	$3\;\text{k}\Omega ,\frac{1}{4},W$	2	$ 0.022	$ 0.044	Mercadolibre	Other
Resistor	10Ω,5,W	2	$ 0.90	$ 1.80	Mercadolibre	Other
Capacitor	100 nF	2	$ 0.77	$ 1.54	Mercadolibre	Polymer
Transistor	IRF640	2	$ 1.14	$ 2.28	Mercadolibre	Semiconductor
Transistor	TIP122	2	$ 0.93	$ 1.86	Mercadolibre	Semiconductor
Transistor	TIP127	2	$ 1.05	$ 2.10	Mercadolibre	Semiconductor
Operational Amplifier	TL081	2	$ 0.41	$ 0.82	Mercadolibre	Semiconductor
Rheostat	100kΩ	1	$ 0.10	$ 0.10	Mercadolibre	Other
Socket	8-pin socket	2	$ 0.043	$ 0.086	Mercadolibre	Other
Terminal	2-pin terminal	1	$ 0.11	$ 0.11	Mercadolibre	Other
Terminal	3-pin terminal	2	$ 0.15	$ 0.30	Mercadolibre	Other
Arduino	Arduino Mega 2560	1	$ 16.13	$ 16.13	Mercadolibre	Other
PCB	Manufactured PCB	1	$ 12.91	$ 12.91	CircuitosFenix	Other
PCB	Manufactured PCB	1	$ 0.55	$ 0.55	Mercadolibre	Other
Fiber	2mm×50cm carbon fiber rod	1	$ 3.00	$ 3.00	Mercadolibre	Composite
Power Source	ATX 750 W power supply Unitec 24-pin PC	1	$ 15.12	$ 15.12	Mercadolibre	Other
Spring	130N/mspring.	2	$ 0.55	$ 1.10	Almacenes todo tornillo	Metal
PLA	Pinion	1	$ 1.15	$ 1.15	Printomics 3D	Polymer
PLA	Rack	2	$ 1.15	$ 3.00	Printomics 3D	Polymer
PLA	Support base	1	$ 4.50	$ 4.50	Printomics 3D	Polymer
PLA	Top cover	1	$ 2.50	$ 2.50	Printomics 3D	Polymer
PLA	Bottom cover	1	$ 2.50	$ 2.50	Printomics 3D	Polymer
PLA	Encapsulation	1	$ 6.00	$ 6.00	Printomics 3D	Polymer
PLA	Indicator	1	$ 1.50	$ 1.50	Printomics 3D	Polymer
Screw	M3 × 6 mm screw	8	$ 0.044	$ 0.176	La Casa del Tornillo	Metal
Washer	M3 Washer	16	$ 0.022	$ 0.352	La Casa del Tornillo	Metal
Nut	M3 Nut	8	$ 0.022	$ 0.176	La Casa del Tornillo	Metal
Shaft	1/8 × 1/2 Rivet	1	$ 0.022	$ 0.022	La Casa del Tornillo	Metal

Instructions for building the device are detailed in Section 5.

## 5 Build instructions

**Fig. 13:**
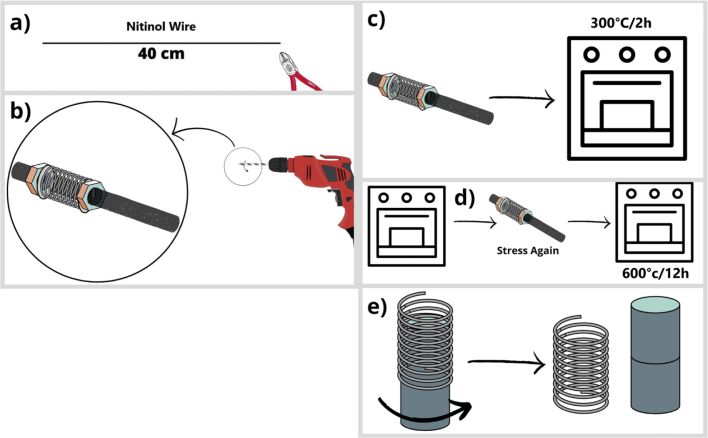
Manufacturing SMA actuator.

To manufacture SMA type actuators, you must follow the following instructions to obtain a functional actuator:

• Cut the Nitinol wire to approximately 40 cm in length (Fig. 13a).
• Using a 3/8 worm screw and a drill, manufacture the actuator as shown in Fig. 13b.
• Once the actuator is ready, you will heat it for 2 h at 300 degrees Celsius (Fig. 13c).
• After the actuator has cooled down, the spring is re-tensioned to generate greater compression, and then heated at 600 degrees Celsius for 12 h (Fig. 13d).
• After the actuator has cooled down following the heating process, remove it from the worm screw (Fig. 13e).

**Fig. 14:**
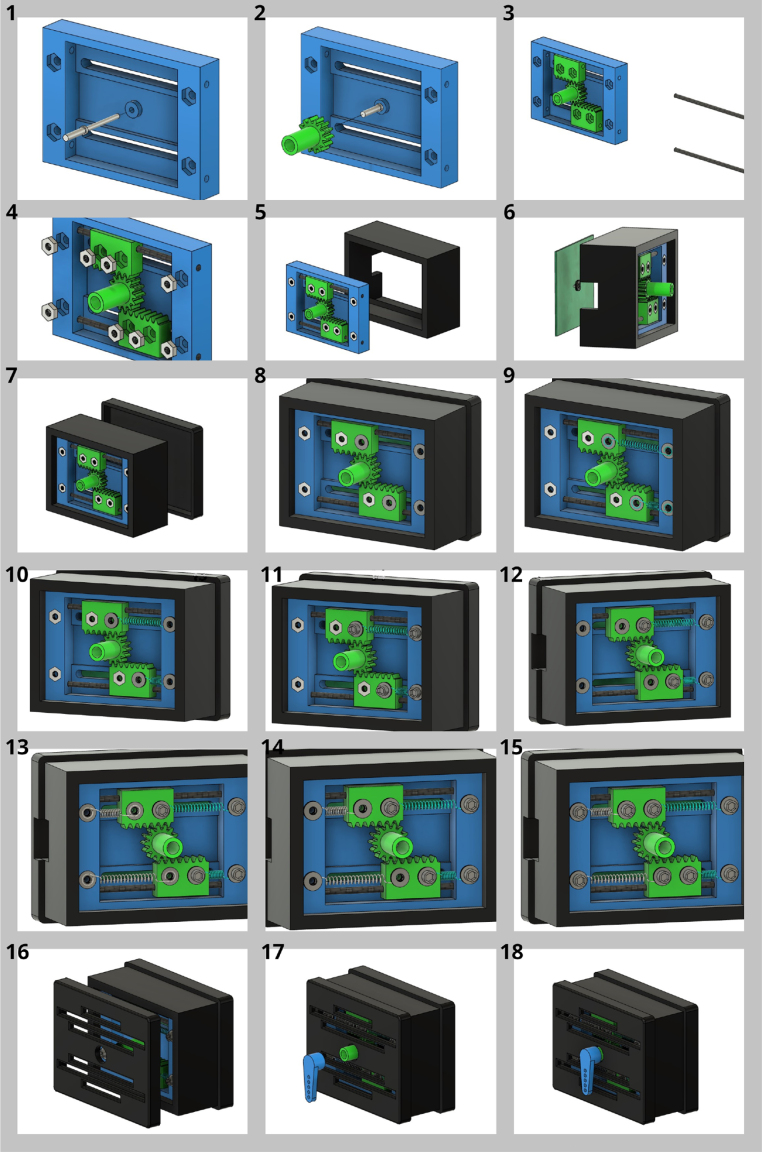
Construction Plans. Sub-figures 1–18 show step by step views of the construction.

The correct operation of the Servomechanism requires the following operation before using the device (Fig. 10):

• Follow the instructions shown in Fig. 14 for the correct construction of the servo mechanism.
• Connect the device to the control system at the corresponding ports for the rheostat and SMA actuators.
• To power the control plant, it is recommended to use a DC power supply with dual polarity, which means it provides both +12 V and −12 V. You should connect the 5 V input of the power supply to the plant, making sure to also connect the corresponding grounds. Then, proceed to connect the PWM inputs of the Arduino to the plant, making sure once again to connect the corresponding grounds. Likewise, connect the analog input of the Arduino to the plant and make sure to connect the corresponding grounds. It is important to verify that all connections are properly made and ensure that the polarity of the power supply is correct.

**Fig. 15:**
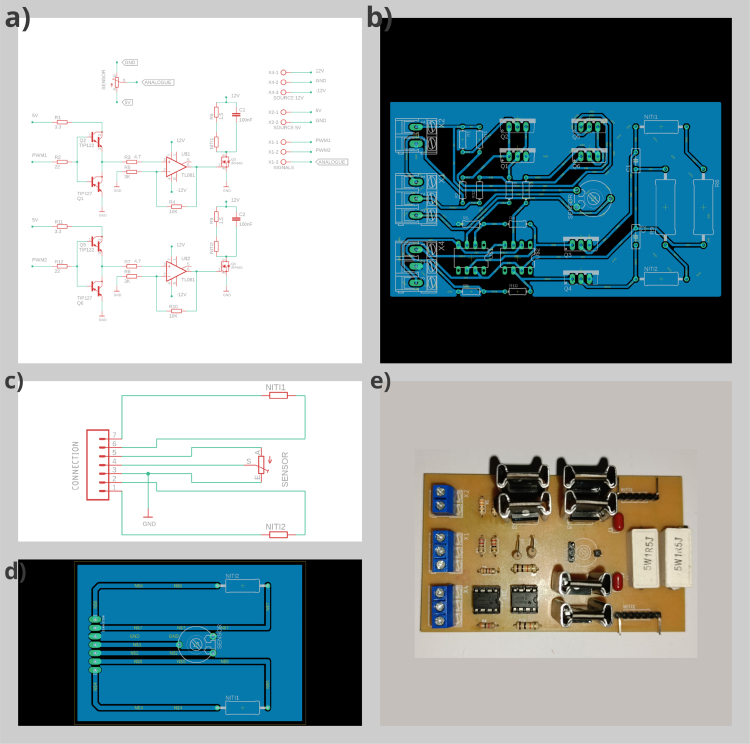
(a) Control plant, (b) Control plant PCB in Eagle, (c) Connection SMA, (d) Connection SMA PCB in Eagle, (e) Manufactured PCB.

## 6 Operation instructions

After the servo-mechanism is manufactured and correctly connected, follow the instructions below to verify its operation:

• Connect the Arduino to the computer via the USB port.
• Open the Code folder and run the Final.ino file.
• Once the code is open, search for the setSetpoint(0) function to set the angle you want the system to move.
• Finally, upload the program to the Arduino.

The characterization and validation results of the device are presented below.

## 7 Validation and characterization

After manufacturing the prototype and verifying that both the mechanical and electronic systems function correctly individually, the first tests of the complete system are carried out.

The central component of the prototype’s mechanical system is the selected Niti alloy. After prolonged use in extreme conditions, the material maintains all its properties without showing signs of fatigue, and the cooling and heating speed remains stable. The deformed length during heating does not vary with use, and the initial length remains constant. The only drawback is the cooling time of the alloy, but this has been reduced by applying pressure in the same direction of the shape recovery. 26 MPa has been applied, exceeding the theoretical 25 MPa, which has reduced the maximum time of the 0.375 mm SMA wire to intervals of 0.6 to 1 s, depending on the deformation. These results are very promising and motivate further research to determine which type of spring is most suitable for increasing the cooling speed.

The other components of the mechanical system, consisting of a shaft, a pinion, and a rack, have been easily mounted and integrated into the structure. Friction and weight of the elements have been minimized. The spring used has performed well and has functioned within its elastic limits, without presenting permanent deformations or changes in its properties. In addition, the PLA used to manufacture the structure has been the ideal material for producing the parts.

Regarding the electronic system, the current control plant for two nitinol SMA wires maintains perfect current regulation and operates smoothly with nominal values of 1.2 A, which is the maximum current provided by the control plant with a 12 V power supply. The detailed tests in the previous chapter have been successful, and the circuit is ready to conduct tests with a dual system. The only issue that arises is the heating of the transistors. Although the heat sinks used have been adequate, they operate at the limit of their capacity. The sensor used behaves correctly within its limitations but introduces a high level of noise. The Arduino is a board that offers outstanding performance for controlling the actuators, and its frequency of 300 kHz is sufficient.

**Fig. 16:**
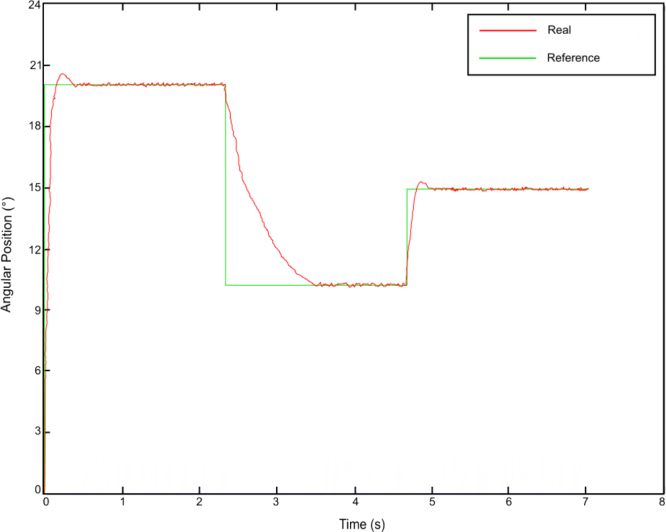
Response of the system with PID control.

The device has been programmed to move the indicator to three specific angles, corresponding to rotations of 10°, 15°, and 20°, and hold it in each position for several seconds. The trajectory graphs of the nitinol’s consecutive heating and cooling can be observed in Fig. 16.

From the previous figure, it can be seen that the response of the system with proportional control adjusts correctly to the reference position. Based on this graph, the fundamental factors that influence the behavior of the actuator are analyzed.

**Fig. 17:**
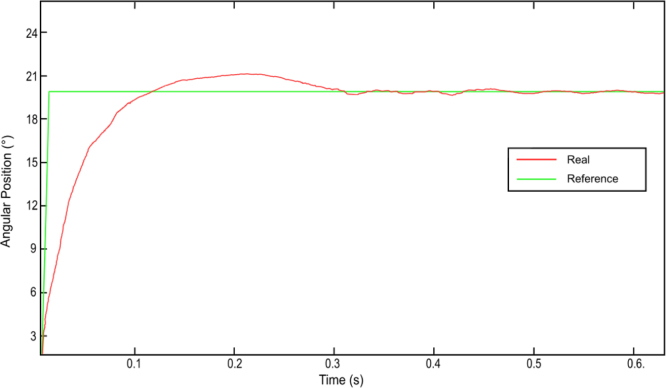
Response time for a 4% deformation.

For an applied current of 1.2 A and a maximum diameter SMA wire, it has been observed that the nitinol’s response time to complete the austenitic transformation until the indicator rotates 20° was approximately 0.25 s (see Fig. 17). This rotation corresponds to an approximate 4% deformation achieved by the nitinol SMA wire.

It is important to highlight that the obtained time is higher than the theoretical value provided by the manufacturer, which is 0.001 s. This time could be reduced if a current peak higher than the nominal current of 1.2 A that the SMA wire supports were applied.

**Fig. 18:**
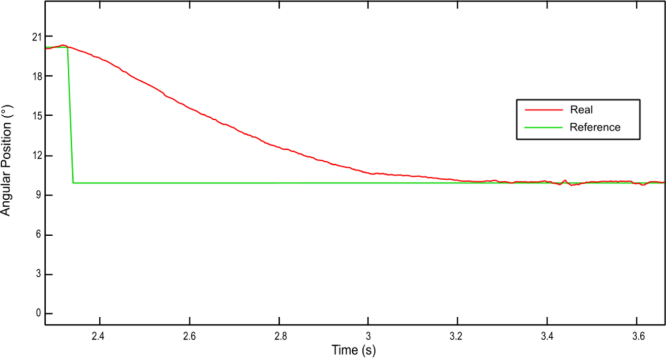
Cooling process. Shape recovery during the martensitic transformation.

During the heating phase, precise control can be performed to ensure the system’s speed and minimum error. For the SMA wire used, the theoretical cooling time is 2 s. However, it has been experimentally observed that the martensitic transformation process takes place in approximately 0.8 s (see Fig. 18). This increase in the cooling speed is due to the traction exerted by the spring. Although this time is shorter than the theoretical value, it is still too high. It will be necessary to study different solutions to reduce it, such as cooling with a thermoconductive liquid or forced air flow. Finally, the conclusions are presented in the following section.

## 8 Conclusions

The development of an actuator using an SMA alloy as an alternative option to conventional systems is feasible, but there are still many challenges to be overcome. The limited research in this field and the few prototypes developed so far have significant limitations. However, the prototype built in this section has shown promise, although it also revealed problems in the electronic design and control of the system. Further research and improvements are necessary to develop viable and effective SMA-based actuation systems that can compete with conventional systems. The successful design and testing of the prototype is a good starting point for future investigations, but challenges such as improving the electronic circuit and control signal require simple solutions that do not overly complicate the system. Only then can SMA materials truly become a viable alternative as an actuator. Nitinol-based systems have no wearing parts such as bearings or gears, allowing for long life. Additionally, the inherent flexibility and lightweight nature of Nitinol wires enable their integration into intricate designs with fewer space constraints compared to bulkier electric motors. This advantage is especially beneficial in applications requiring precise and delicate movements, such as medical devices or micro-robotics.

## Ethics statements

This work did not involve any human or animal subjects, nor data from social media platforms.

## CRediT authorship contribution statement

**Santiago Zuñiga:** Writing – original draft. **Diego Bravo:** Investigation. **Carlos Rengifo:** Writing – review & editing.

## Declaration of competing interest

The authors declare that they have no known competing financial interests or personal relationships that could have appeared to influence the work reported in this paper.
